# Postoperative Dry Eye Following Anterior Lamellar Recession Without Tarsal Fracture Versus Marginal Rotation With Tarsal Fracture in the Treatment of Cicatricial Entropion: A Comparative Study

**DOI:** 10.7759/cureus.97411

**Published:** 2025-11-21

**Authors:** Walid M Abdalla, Eman N Sultan

**Affiliations:** 1 Ophthalmology, Orbit Eye Center, Dubai Healthcare City, Dubai, ARE; 2 Ophthalmology, Community Health and Eyecare Limited (CHEC) New Cross, London, GBR

**Keywords:** anterior lamellar recession, cicatricial entropion, conjunctival scarring, trachoma, trichiasis

## Abstract

Objective

This study aimed to compare the postoperative dry eye parameters following anterior lamellar recession without tarsal fracture versus tarsal fracture with marginal rotation in patients treated for cicatricial entropion.

Methods

This retrospective cohort study included adult patients diagnosed with primary or recurrent cicatricial entropion who underwent either anterior lamellar recession without tarsal fracture or tarsal fracture with marginal rotation. A comprehensive ocular assessment was performed pre- and postoperatively, including evaluation of tear film meniscus level, tear break-up time (TBUT), corneal fluorescein staining, Schirmer test, and Ocular Surface Disease Index (OSDI) questionnaire. In addition, the recurrence of entropion and associated symptoms was assessed.

Results

A total of 50 eyes were included in the study. Group A consisted of 25 patients who underwent anterior lamellar recession without tarsal fracture, with a mean age of 59.2±5.1 years. Group B consisted of 25 patients who underwent marginal rotation with tarsal fracture, with a mean age of 59.3±5.0 years. Both groups showed different degrees of deterioration of tear film stability after surgery. Group A showed significantly better results regarding the tear film meniscus level (p<0.001), TBUT (p=0.009), and OSDI (p=0.002) compared to group B. Meanwhile, the two groups were comparable regarding the recurrence rate (p=0.384).

Conclusion

Both procedures have a comparable success rate. However, anterior lamellar recession without tarsal fracture is associated with better results in terms of tear film stability and quality in the postoperative period.

## Introduction

Dry eye is a multifactorial condition characterized by a loss of tear film homeostasis, with co-occurring manifestations such as eye discomfort, visual disturbances, and tear film instability [1,2]. There is a paucity of data in the literature regarding the rate of dry eye following surgical correction of cicatricial entropion. However, a considerable proportion of patients is expected to suffer from post-surgery dry eye. Several factors may interact to contribute to dry eye following surgical correction of cicatricial entropion; some of these factors are produced by entropion itself, while others are related to the surgery. Cicatricial entropion induces chronic inflammation and conjunctival scarring, resulting in disruption of the normal lid margin anatomy [3]. In addition, these changes may cause the mucosal tissue to overlay the orifices of the meibomian, which is known as conjunctivalization of the lid margin [4]. Consequently, secretions from meibomian glands (MGs) are reduced, leading to disruption of the tear film [3]. The surgical manipulation itself poses additional mechanical trauma to the ocular surface and eyelid, which can exacerbate inflammation and lead to disruption of the tear film [5,6].

Various surgical techniques have been proposed to correct cicatricial entropion and reduce the incidence of recurrence due to fibrosis. This study aimed to compare the postoperative dry eye parameters following anterior lamellar recession without tarsal fracture versus tarsal fracture with marginal rotation in patients treated for cicatricial entropion.

## Materials and methods

Study design

This retrospective cohort study adheres to the tenets of the Declaration of Helsinki. Confidentiality of patient information was maintained by assigning a unique code to each patient, known only to the investigators, while data sheets were kept anonymous. Records of patients at the Cairo Teaching Hospitals during the period from 2021 to 2023 were reviewed.

Eligibility criteria

The study included all adult patients diagnosed with primary or recurrent cicatricial entropion who underwent either anterior lamellar recession without tarsal fracture or tarsal fracture with marginal rotation. We excluded patients younger than 18 years of age at the time of surgery or diagnosed with any type of entropion other than cicatricial entropion.

Data collection

Preoperatively, data collected included medical history (age, sex, and history of previous surgery for entropion); ocular examination of the eyelid, cornea, and conjunctiva; evaluation of tear film meniscus level, tear break-up time (TBUT), corneal fluorescein staining, and Schirmer's test; and the Ocular Surface Disease Index (OSDI) [7]. Postoperatively, patients were examined on the first postoperative day and then at one week, one month, three months, and six months. All patients underwent evaluation of tear film meniscus level, TBUT, corneal fluorescein staining, Schirmer's test, and were administered the OSDI questionnaire at six months after surgery.

Surgical technique and outcomes

A total of 50 eyes were included in the study. Group A consisted of 25 patients who underwent anterior lamellar recession without tarsal fracture. Group B consisted of 25 patients who underwent tarsal fracture with marginal rotation. The choice of procedure was based on the surgeon’s preference and experience, and independent of the patient’s condition or severity of entropion. Both procedures were performed under local anaesthesia by local infiltration of 2% lidocaine with 1:100,000 epinephrine. 

In group A, a lid crease incision was made using a #11 blade, followed by blunt dissection above the tarsus to release any scar tissue till reaching 4 mm above the lash line. The lid margin was split and recessed superiorly, and 5/0 vicryl everting sutures were used to evert the lid margin and were tied 4 mm above the lash line. In group B, a traction suture was placed, and the tarsus was then completely incised from the conjunctival side in the sulcus subtarsalis, extending from the nasal to the temporal ends of the tarsus using a #11 blade. Five-zero (5-0) Vicryl everting sutures were passed from the conjunctival side to the cutaneous side and were tied 4 mm above the lash line.

Postoperative treatment for both procedures included topical antibiotics for six weeks, while systemic antibiotics and anti-inflammatory agents were only prescribed for patients with severe postoperative eyelid edema or stitch infection. Primary outcomes included postoperative evaluation of tear meniscus level, TBUT, corneal fluorescein staining, Schirmer's test, and OSDI, while secondary outcomes included dry eye symptoms and recurrence rate of entropion.

Statistical analysis

Analyses were performed using the R Statistical Language version 4.4.1 (R Foundation for Statistical Computing, Vienna, AUT) and the packages ggstatsplot (version 0.12.4) and gtsummary (version 2.0.0). The distribution of continuous numerical variables was assessed using the Shapiro-Wilk test and Q-Q plots. Continuous variables that followed a normal distribution were summarized using the mean, standard deviation, and range. Variables that did not follow normal distribution were summarized using the median, interquartile range (IQR) expressed as 25th to 75th percentiles, and range. Categorical variables were summarized as counts and frequencies. Comparisons between the two groups were made using either the two-sample T-test or the Wilcoxon rank-sum test (for normally and abnormally distributed variables, respectively), whereas within-group comparisons between preoperative and postoperative measurements were made using the paired T-test or the Wilcoxon signed-rank test (for normally and abnormally distributed variables, respectively). The association between the type of surgery and categorical variables was tested using either Pearson’s chi-squared test for independence of observations or Fisher’s exact test. A p-value < 0.05 was considered statistically significant. 

## Results

The present study included 50 patients. The two groups were comparable regarding patients’ age (59.2±5.1 and 59.3±5.0 years, p=0.933). The distribution of sex was similar in the two groups (p>0.999), with a male-to-female ratio of 1:4. In each group, 21 (84%) entropion cases were primary, while four (16%) were recurrent (p>0.999), as shown in Table 1. 

**Table 1:** Characteristics of patients ^1^Two sample T-test; ^2^Pearson’s chi-squared test; ^3^Fisher’s exact test. The data are represented as mean ± SD or n (%). A p-value <0.05 is considered significant.

Variable		Group A (n = 25)	Group B (n = 25)	Test statistic	p-value
Age (year)	Mean ± SD	59.2 ± 5.1	59.3 ± 5.0	-0.084	0.933 ^1^
	Range	50.0 - 68.0	50.0 - 68.0		
Sex, n (%)	Female	20 (80.0%)	20 (80.0%)	0.000	>0.999 ^2^
	Male	5 (20.0%)	5 (20.0%)		
Entropion, n (%)	Primary	21 (84.0%)	21 (84.0%)	-	>0.999 ^3^
	Recurrent	4 (16.0%)	4 (16.0%)		

Before surgery, corneal affection was present in seven (28%) cases in each group. One week after surgery, mild lid edema and mild redness were detected in some patients, while nearly half the patients showed no abnormal findings, with no significant differences between the two groups (p=0.943). At follow-up after surgery, dry eye was detected in 11 (44%) cases of group A patients and 10 cases (40%) of group B patients, with no statistically significant difference (p=0.774) at one, three, and six months after surgery (Table 2).

**Table 2:** Preoperative and postoperative findings in the studied groups ^1 ^Pearson’s chi-squared test. The data are represented as n (%). A p-value <0.05. is considered significant.

Variable		Group A (n = 25)	Group B (n = 25)	Test statistic	p-value ^1^
Preoperative corneal affection, n (%)	Absent	18 (72.0%)	18 (72.0%)	0.000	>0.999
	Present	7 (28.0%)	7 (28.0%)		
First week, n (%)	Normal	12 (48.0%)	13 (52.0%)	0.117	0.943
	Mild lid edema	6 (24.0%)	6 (24.0%)		
	Mild redness	7 (28.0%)	6 (24.0%)		
First months, n (%)	Normal	14 (56.0%)	15 (60.0%)	0.082	0.774
	Dry eye	11 (44.0%)	10 (40.0%)		
Three months, n (%)	Normal	14 (56.0%)	15 (60.0%)	0.082	0.774
	Dry eye	11 (44.0%)	10 (40.0%)		
Six months, n (%)	Normal	14 (56.0%)	15 (60.0%)	0.082	0.774
	Dry eye	11 (44.0%)	10 (40.0%)		

**Figure 1:**
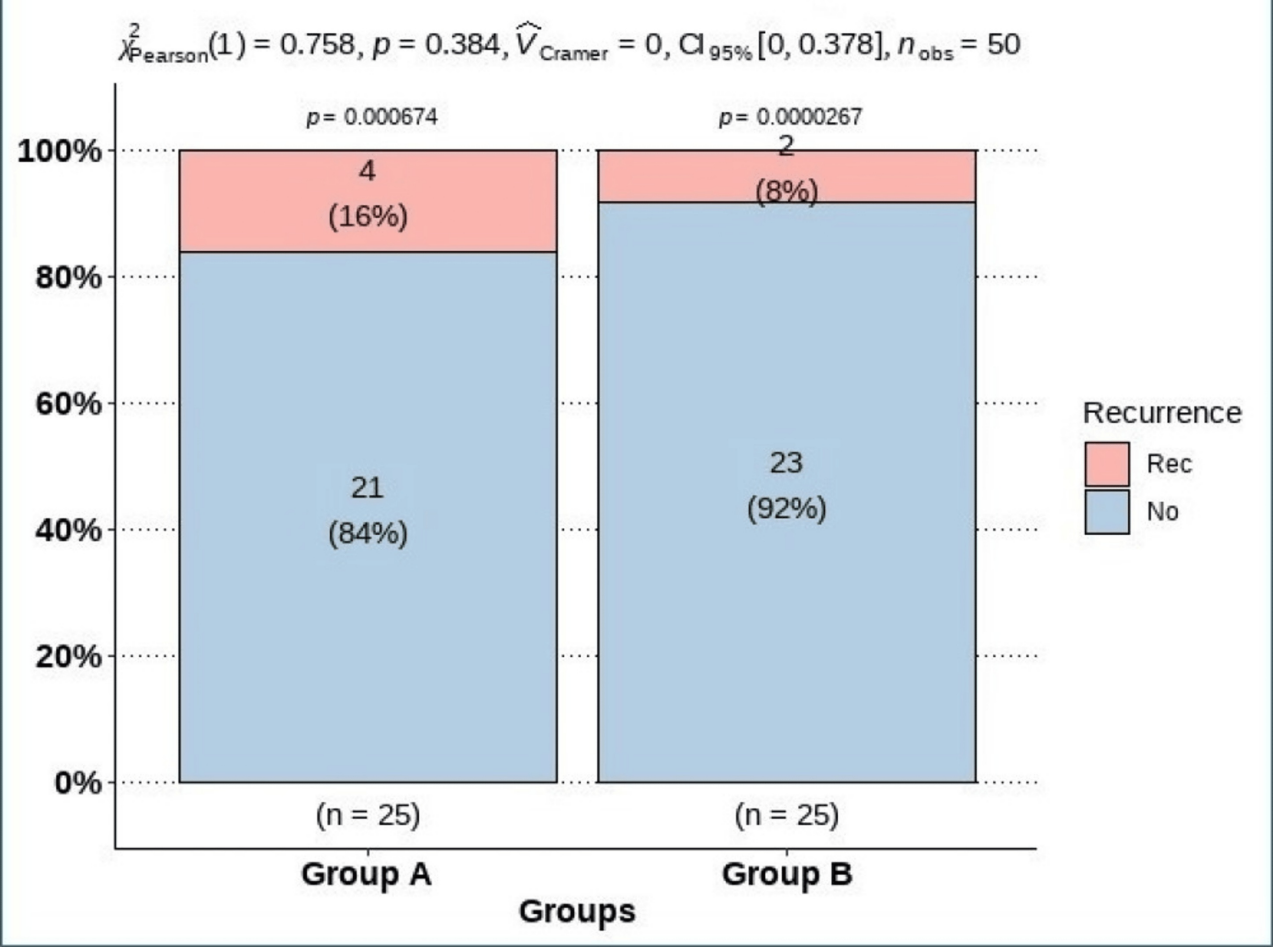
Recurrence during postoperative follow-up The data are represented as n (%). A p-value <0.05 is considered significant.

Comparison of preoperative and postoperative findings in group A patients showed a significant decrease in OSDI after surgery (p<0.001). There was a lack of significant changes in terms of tear film meniscus level (p=0.131), TBUT (p=0.327), and Schirmer test (p=0.894). The percentage of patients with positive corneal fluorescein staining and associated itching, redness, and burning sensation decreased after surgery, while the percentage of patients with a foreign body sensation increased but without reaching statistical significance (all p-values >0.05). As for group B patients, there was a significant decrease after surgery in the values of tear film meniscus level (p<0.001), TBUT (p<0.001), Schirmer test (p<0.001), OSDI (p<0.001), and punctate epithelial erosion (PEE) (p=0.023). Comparison of preoperative findings between group A and group B patients showed a lack of significant differences in all assessed measurements, except that seven (28%) patients in group B had PEE, while none of the group A patients had PEE (p=0.010). After surgery, group B patients showed significantly lower values of tear film meniscus level (p<0.001) and tear breakup time (p=0.009) compared to group A. Meanwhile, the postoperative values of OSDI were significantly higher in group B patients compared to group A (p=0.002) (Table 3). The rate of recurrence was 16% (four cases) and 8% (two cases) in group A and group B patients, respectively, with no statistically significant difference (p=0.384) (Figure 1).

**Table 3:** Within-groups and between-groups comparisons of assessed measurements and manifestations IQR: Interquartile range; OSDI: Ocular Surface Disease Index; PEE: Punctate epithelial erosions; P1: The p-value from tests comparing preoperative and postoperative data in group A; P2: The p-value from tests comparing preoperative and postoperative data in group B; P3: The p-value from tests comparing preoperative data between group A and group B; P4: The p-value from tests comparing postoperative data between group A and group B; ^1^Wilcoxon signed rank test with continuity correction; ^2^Wilcoxon rank sum test; ^3^Paired t-test; ^4^Welch two sample t-test; ^5^McNemar’s chi-squared test with continuity correction;^ 6^Fisher’s exact test; ^7^Pearson’s chi-squared test The data are represented as median (IQR), mean ± SD, or n (%). A p-value <0.05 is considered significant.

Variable		Group A		Group B		Test statistic (P1)	Test statistic (P2)	Test statistic (P3)	Test statistic (P4)
		Preoperative (n=25)	Postoperative (n=25)	Preoperative (n=25)	Postoperative (n=25)				
Tear film meniscus level	Median (IQR)	0.3 (0.2 - 0.4)	0.3 (0.2 - 0.4)	0.3 (0.2 - 0.3)	0.2 (0.2 - 0.2)	9.500 (0.131^1^)	171.000 (<0.001*^1^)	407.500 (0.060^2^)	513.000 (<0.001*^2^)
	Range	0.1 - 0.4	0.1 - 0.4	0.1 - 0.4	0.1 - 0.3				
Tear breakup time	Mean ± SD	10.1 ± 2.6	10.2 ± 2.7	10.8 ± 1.9	8.4 ± 2.1	-1.000 (0.327^3^)	6.928 (<0.001*^3^)	-1.005 (0.320^4^)	2.741 (0.009*^4^)
	Range	5.0 to 15.0	4.0 to 15.0	6.0 to 14.0	5.0 to 12.0				
Corneal fluorescein staining, n (%)	Negative	19 (76.0%)	22 (88.0%)	20 (80.0%)	23 (92.0%)	-1.000 (0.327^3^)	0.800 (0.371^5^)	0.117 (0.733^6,7^)	(>0.999^6,7^)
	Positive	6 (24.0%)	3 (12.0%)	5 (20.0%)	2 (8.0%)				
Schirmer test	Mean ± SD	11.2 ± 2.9	11.2 ± 2.9	11.7 ± 2.9	10.3 ± 1.7	325.000 (0.894^3^)	4.404 (<0.001*^3^)	-0.689 (0.494^4^)	1.374 (0.177^4^)
	Range	5.0 to 16.0	6.0 to 16.0	8.0 to 17.0	8.0 to 13.0				
OSDI	Median (IQR)	14.0 (12.0 - 16.0)	2.0 (1.0 - 3.0)	14.0 (13.0 - 17.0)	6.0 (2.0 - 7.0)	325.000 (<0.001*^1^)	300.000 (<0.001*^1^)	275.500 (0.473^2^)	153.500 (0.002*^2^)
	Range	10.0 - 19.0	0.0 - 4.0	9.0 - 19.0	0.0 - 14.0				
Associated symptoms, n (%)	No significant symptoms	16 (64.0%)	21 (84.0%)	18 (72.0%)	17 (68.0%)	3.200 (0.074^5^)	0.000 (>0.999^5^)	0.368 (0.544^7^)	1.754 (0.185^7^)
	Itchiness	2 (8.0%)	1 (4.0%)	0 (0.0%)	2 (8.0%)	0.000 (>0.999^5^)	0.500 (0.480^5^)	(0.490^6^)	(>0.999^6^)
	FB Sensation	1 (4.0%)	3 (12.0%)	0 (0.0%)	1 (4.0%)	0.500 (0.480^5^)	0.000 (>0.999^5^)	(>0.999^6^)	(0.609^6^)
	Redness	3 (12.0%)	0 (0%)	0 (0.0%)	0 (0.0%)	1.333 (0.248^5^)	-	(0.235^6^)	(>0.999^6^)
	Burning Sensation	3 (12.0%)	1 (4.0%)	0 (0.0%)	0 (0.0%)	0.250 (0.617^5^)	-	(0.235^6^)	(>0.999^6^)
	PEE	0 (0.0%)	0 (0.0%)	7 (28.0%)	0 (0.0%)	-	5.143 (0.023*^5^)	(0.010*^6^)	(>0.999^6^)
	Gritty Sensation	0 (0.0%)	0 (0.0%)	0 (0.0%)	4 (16.0%)	-	2.250 (0.134^5^)	(>0.999^6^)	(0.110^6^)

## Discussion

The present study endeavored to compare the postoperative dry eye parameters following anterior lamellar recession without tarsal fracture versus tarsal fracture with marginal rotation in patients treated for cicatricial entropion. It included 50 patients (25 patients in each group). There was a female predominance, with a male-to-female ratio of 1:4 in each group. This is in alignment with previous studies that reported a higher percentage of females among patients with cicatricial entropion, with the male-to-female ratio varying between approximately 1:4 [8] and 2:3 [9,10]. This can be explained by the fact that females tend to have smaller tarsal plates than males, making them more susceptible to developing entropion [11].

In the current study, the rates of postoperative dry eye were similar in the two groups (11 cases (44%) and 10 cases (40%) in groups A and B, respectively), suggesting no significant association between dry eye and the type of surgery (p=0.774). The persistence or development of dry eye after surgical correction of entropion may be partially attributed to changes in the anatomy and function of the MGs, as described in previous studies [12-14]. In cicatricial entropion, keratinization of the lid margin often occurs [15,16], leading to blockage of the MG orifices and accumulation of their secretions. Trapped secretions lead to inflammation, cystic dilatation, and eventually atrophy of the acini [12,17]. Surgical correction can reverse these effects by restoring the position of the MG openings, allowing the release of trapped secretions. The beneficial effects of entropion repair on dry eye symptoms have been reported to last up to five months after surgery [18].

In the current study, both groups showed different degrees of deterioration of tear film stability after surgery. The differences between preoperative and postoperative values of tear film meniscus level, TBUT, and Schirmer test were not significant in group A, while the differences were significant in group B. Accordingly, group B had significantly lower values of tear film meniscus level (p<0.001) and TBUT (p=0.009), as well as higher values of OSDI compared to group A (p=0.002). These findings indicate that the parameters of the tear film in patients of group A did not deteriorate after surgery. On the other hand, group B showed more worsening in the quality of the tear film. Tear breakup time reflects the quality and stability of the tear film. Disruption of tear film stability may occur in cicatricial entropion due to ongoing chronic inflammation and cicatricial changes that impede the secretion of water and mucin components [19,20].

Our results are partially consistent with those of previous studies comparing different techniques for the treatment of cicatricial entropion. Monga et al. [21] compared three corrective surgical techniques for trachomatous entropion: terminal tarsal rotation after transverse tarsotomy (Kettesy's procedure), tarsal rotation with tarsoconjunctival advancement (Collin's modification of Trabut's procedure), and anterior lamellar repositioning with lid margin split and tarsal wedge resection. They found that postoperative changes in TBUT and the Schirmer test were non-significantly more favorable after Kettesy's procedure, followed by Collin's modification of Trabut's procedure, while anterior lamellar repositioning showed changes that were less favorable compared to the other procedures.

Abd-Elbary et al. [10] compared the tarsal fracture technique and anterior lamellar reposition with the grey line split technique for the correction of cicatricial entropion. They found that both techniques resulted in a significant decrease in postoperative TBUT compared to preoperative values, but the differences between groups did not reach statistical significance. Some studies showed results contradictory to our findings. Daifalla et al. [20] found a highly significant improvement in TBUT and the Schirmer test after tarsal rotation surgery compared to preoperative levels. They also reported a significant decrease in the percentage of positive fluorescein staining after tarsal rotation surgery compared to preoperative staining.

Several confounding factors may have contributed to the observed differences between studies. The differences in the underlying cause of entropion, as well as the degree of severity of entropion between studies, may lead to variations in the outcomes studied. In addition, some studies may have included patients with mixed involutional and cicatricial entropion. Another potential contributing factor is the extent of surgical intervention, as extensive manipulation may adversely affect the patency and positioning of MGs, leading to worsening of dry eye symptoms in the postoperative period. The extent of surgical manipulation is influenced by factors such as the condition of the operated eye, the chosen surgical technique, the duration of the procedure, and the experience of the surgeon. Monga et al. [21] suggested that the Kettesy procedure may provide slightly better outcomes, as it typically involves a shorter operative time and less extensive dissection [22] and preserves the anatomy of the upper eyelid as well as the integrity of MGs [23].

Recurrence rates after surgical correction are high, and many patients require multiple procedures during their lifetime [24]. We found that the recurrence rate of entropion was slightly higher in group A compared to group B (16% (four cases) and 8% (two cases), respectively), but this difference did not reach statistical significance (p=0.384). This result is comparable to the recurrence rates reported in earlier studies following anterior lamellar recession [25,26]. For the tarsal fracture procedure, the reported rates varied from 26% [27] to 6% [28]. The variation in recurrence rates may be due to differences in the underlying cause of scarring entropion as described in previous studies [25,26]. In addition, anterior lamellar recession without tarsal fracture may not sufficiently address or resolve the cicatricial force caused by scarring and contracture in the posterior lamella (the tarsus and conjunctiva), so the inward rotation may recur after surgery [29,30]. 

The present study has several strengths, including the assessment of changes in tear film stability along with patient-reported outcomes related to dry eye. However, the study had some limitations because the follow-up period was only six months. Therefore, future studies should evaluate long-term outcomes at different follow-up time points. In addition, the underlying cause of the scarring entropion in our patients was not recorded in the cohorts studied.

## Conclusions

Both anterior lamellar recession without tarsal fracture and marginal rotation with tarsal fracture were effective in managing cicatricial entropion, with comparable recurrence rates observed in this cohort. However, meaningful differences emerged in postoperative ocular surface parameters. Patients who underwent anterior lamellar recession demonstrated better preservation of tear film stability compared to those who underwent tarsal fracture with marginal rotation. In contrast, the tarsal fracture technique was associated with greater postoperative deterioration of tear film quality, which may contribute to dry eye symptoms during the recovery period. These findings suggest that while both procedures remain viable surgical options, anterior lamellar recession without tarsal fracture may be preferable for patients at higher risk of postoperative dry eye or in whom tear film preservation is a priority. Further prospective studies with larger sample sizes and longer follow-up are warranted to validate these outcomes and guide individualized surgical decision-making.

## References

[1] Tsubota K, Pflugfelder SC, Liu Z (2020). Defining dry eye from a clinical perspective. Int J Mol Sci.

[2] Kojima T, Dogru M, Kawashima M, Nakamura S, Tsubota K (2020). Advances in the diagnosis and treatment of dry eye. Prog Retin Eye Res.

[3] Lucena A, Akaishi PM, Rodrigues Mde L, Cruz AA (2012). Upper eyelid entropion and dry eye in cicatricial trachoma without trichiasis. Arq Bras Oftalmol.

[4] Kemp EG, Collin JR (1986). Surgical management of upper lid entropion. Br J Ophthalmol.

[5] Lai LY, Springs CL, Burgett RA (2015). Surgical management of dry eyes. Dry Eye: A Practical Approach.

[6] Fan W, Rokohl AC, Guo Y, Heindl LM (2021). Ocular surface and tear film changes after eyelid surgery. Ann Eye Sci.

[7] Schiffman RM, Christianson MD, Jacobsen G, Hirsch JD, Reis BL (2000). Reliability and validity of the Ocular Surface Disease Index. Arch Ophthalmol.

[8] Elessawy KB, Elnagar AM, Nasr HE, Abdelbaky SH (2021). Anterior lamellar recession with and without blepharoplasty in upper eyelid cicatricial entropion. J Egypt Ophthalmol Soc.

[9] Awny I (2020). Anterior Lamellar recession versus tarsal fracture for management of recurrent cicatricial upper lid entropion: a randomized comparative study. Egypt J Clin Ophthalmol.

[10] Abd-Elbary DF, Shabana RR, Shalaby OE-s, El-Desouky MA (2023). Evaluation of meibomian gland dysfunction before and after surgical correction of cicatricial entropion of the upper eye lid. J Adv Med Med Res.

[11] Bashour M, Harvey J (2000). Causes of involutional ectropion and entropion — age-related tarsal changes are the key. Ophthalmic Plast Reconstr Surg.

[12] Al-Rajhi AA, Hidayat A, Nasr A, Al-Faran M (1993). The histopathology and the mechanism of entropion in patients with trachoma. Ophthalmology.

[13] Eom Y, Lee JS, Kang SY, Kim HM, Song JS (2013). Correlation between quantitative measurements of tear film lipid layer thickness and meibomian gland loss in patients with obstructive meibomian gland dysfunction and normal controls. Am J Ophthalmol.

[14] Jung JW, Park SY, Kim JS, Kim EK, Seo KY, Kim TI (2016). Analysis of factors associated with the tear film lipid layer thickness in normal eyes and patients with dry eye syndrome. Invest Ophthalmol Vis Sci.

[15] Kuckelkorn R, Schrage N, Becker J, Reim M (1997). Tarsoconjunctival advancement: a modified surgical technique to correct cicatricial entropion and metaplasia of the marginal tarsus. Ophthalmic Surg Lasers.

[16] Nelson JD, Shimazaki J, Benitez-del-Castillo JM, Craig JP, McCulley JP, Den S, Foulks GN (2011). The international workshop on meibomian gland dysfunction: report of the definition and classification subcommittee. Invest Ophthalmol Vis Sci.

[17] Siah WF, Boboridis K, Tan P, Litwin AS, Daya SM, Malhotra R (2019). Meibomian gland inversion: under-recognized entity. Acta Ophthalmol.

[18] Yang MK, Sa HS, Kim N, Jeon HS, Hyon JY, Choung H, Khwarg SI (2022). Quantitative analysis of morphological and functional alterations of the meibomian glands in eyes with marginal entropion. PLoS One.

[19] Moudgil SS, Singh M, Parmar IP, Khurana AK (1986). Study of tear film flow and break up time (BUT) in trachoma. Acta Ophthalmol (Copenh).

[20] Daifalla AE, Hassan GR, Faramawi HM (2024). Clinical evaluation of changes in ocular surface integrity after upper eye lid entropion surgery. Benha J Appl Sci.

[21] Monga P, Gupta VP, Dhaliwal U (2008). Clinical evaluation of changes in cornea and tear film after surgery for trachomatous upper lid entropion. Eye (Lond).

[22] Dhaliwal U, Monga PK, Gupta VP (2004). Comparison of three surgical procedures of differing complexity in the correction of trachomatous upper lid entropion: a prospective study. Orbit.

[23] Bi YL, Zhou Q, Xu W, Rong A (2009). Anterior lamellar repositioning with complete lid split: a modified method for treating upper eyelids trichiasis in Asian patients. J Plast Reconstr Aesthet Surg.

[24] Lifton J, Meer E, Vagefi MR (2023). Surgical management of cicatricial entropion. Plastic Surgery of the Lower Eyelids.

[25] Singh S, Basu S, Jakati S (2023). Cicatricial entropion in chronic cicatrizing conjunctivitis : potential pathophysiologic mechanisms and long-term outcomes of a modified technique. Ophthalmic Plast Reconstr Surg.

[26] Anklesaria V, Ogbu N, Singh S (2025). Long-term outcomes of eyelash-sparing surgical technique for severe segmental cicatricial entropion. Eur J Ophthalmol.

[27] Pombejera F, Tirakunwichcha S (2011). Tarsal fracture operation in cicatricial entropion. J Med Assoc Thai.

[28] Kersten RC, Kleiner FP, Kulwin DR (1992). Tarsotomy for the treatment of cicatricial entropion with trichiasis. Arch Ophthalmol.

[29] Elder MJ, Collin R (1996). Anterior lamellar repositioning and grey line split for upper lid entropion in ocular cicatricial pemphigoid. Eye (Lond).

[30] Diab MM, Allen RC (2021). Recurrent upper eyelid trachomatous entropion repair: long-term efficacy of a five-step approach. Eye (Lond).

